# Profile Changes in the Soil Microbial Community When Desert Becomes Oasis

**DOI:** 10.1371/journal.pone.0139626

**Published:** 2015-10-01

**Authors:** Chen-hua Li, Li-song Tang, Zhong-jun Jia, Yan Li

**Affiliations:** 1 State Key Laboratory of Desert and Oasis Ecology, Xinjiang Institute of Ecology and Geography, Chinese Academy of Sciences, Urumqi, Xinjiang, China; 2 State Key Laboratory of Soil and Sustainable Agriculture, Nanjing Institute of Soil Science, Chinese Academy of Sciences, Nanjing, China; University of Oklahoma, UNITED STATES

## Abstract

The conversion of virgin desert into oasis farmland creates two contrasting types of land-cover. During oasis formation with irrigation and fertilizer application, however, the changes in the soil microbial population, which play critical roles in the ecosystem, remain poorly understood. We applied high-throughput pyrosequencing to investigate bacterial and archaeal communities throughout the profile (0–3 m) in an experimental field, where irrigation and fertilization began in 1990 and cropped with winter wheat since then. To assess the effects of cultivation, the following treatments were compared with the virgin desert: CK (no fertilizer), PK, NK, NP, NPK, NPKR, and NPKM (R: straw residue; M: manure fertilizer). Irrigation had a greater impact on the overall microbial community than fertilizer application. The greatest impact occurred in topsoil (0–0.2 m), e.g., *Cyanobacteria* (25% total abundance) were most abundant in desert soil, while *Actinobacteria* (26%) were most abundant in oasis soil. The proportions of extremophilic and photosynthetic groups (e.g., *Deinococcus-Thermus* and *Cyanobacteria*) decreased, while the proportions of R-strategy (e.g., *Gammaproteobacteria* including *Xanthomonadales*), nitrifying (e.g., *Nitrospirae*), and anaerobic bacteria (e.g., *Anaerolineae*) increased throughout the oasis profile. Archaea occurred only in oasis soil. The impact of fertilizer application was mainly reflected in the non-dominant communities or finer taxonomic divisions. Oasis formation led to a dramatic shift in microbial community and enhanced soil enzyme activities. The rapidly increased soil moisture and decreased salt caused by irrigation were responsible for this shift. Furthermore, difference in fertilization and crop growth altered the organic carbon contents in the soil, which resulted in differences of microbial communities within oasis.

## Introduction

Soil microbes play fundamental roles in soil biogeochemical processes. Land use and management have significant effects on the microbial community structure and function, and this may subsequently influence soil quality and ecological stability [1]. Agricultural use of natural soils is considered to have produced negative effects [2]. For example, cultivation on forest or meadow soils can change the microbial community structure and rapidly decrease soil organic carbon (SOC) [3–5], and the overuse of nitrogen (N) fertilizer can result in a decreasing microbial diversity and loss of SOC [6–8]. However, there are also management practices that are considered to have positive effects on microbial diversity, activity and SOC sequestration, e.g., the combined application of chemical and organic fertilizer [9–10].

Arid lands account for nearly 30% of global terrestrial ecosystems. There is an increasing need for large-scale cultivation in desert regions to feed human populations. When virgin desert is cultivated into oasis farmland, two contrasting landscape or land-cover types are formed within the arid land. Due to drought and low nutrient levels, irrigation and fertilization are necessary to ensure crop yields and oasis formation. The large quantity of water and fertilizer applications and the dramatically increased primary productivity inevitably leads to remarkable changes in the soil. Furthermore, the impact is not limited to the topsoil where the water and fertilizer is directly applied, but the deep soil which is impacted by leached substances and altered root systems [11]. These changes can alter the abundance and composition of the microbial community and its function, and in turn significantly influence the dynamics of ecological processes in the topsoil and deep soil [12–13]. Previous studies have suggested that cultivation in deserts may lead to the disappearance of some extremophilic bacterial groups, but would promote bacterial diversity and plant health [14]. However, because of the very low levels of cultivable microorganisms and the methodological limitations in microbial ecology, our knowledge of desert soil micro-organisms remains fragmentary. Furthermore, more comprehensive studies of a wider range of soils and management practices are needed to fully understand the shift in microbial communities during oasis formation.

The methods currently used to study soil microbial communities are mainly based on fingerprint technology [15] and soil biochemical techniques [16]. However, these methods do not provide sufficient information to comprehensively resolve the phylogenetic and toxicological responses of microbial communities to changes in environmental conditions. The high-throughput pyrosequencing technique is based on high resolution sequencing analysis. It can obtain large amounts of information and provide more meaningful comparisons of microbial communities that may be affected by different management practices [17–18].

This study was conducted at the southern periphery of the Gurbantonggut Desert, which is characterized by dryness, high soil salinity, low nutrient levels, and intense radiation [19]. As a typical arid region, irrigated farmland is the prevailing land-use type in agriculture. Our preceding research showed that cultivation in the desert significantly changed soil properties and increased microbial biomass [20]. In the current study, we compared the oasis soils receiving different fertilizer applications with the virgin desert to study the effects of cultivation on soil microbial communities, using high-throughput pyrosequencing. The objective of the study was to assess the changes in microbial communities throughout the soil profile during oasis formation, and to evaluate any possible linkages between these communities and soil properties, including enzyme activity. We hypothesized that: (1) cultivation in desert region should result in a remarkable shift in the structure of the microbial community, not only in the topsoil but also in the deep soil. (2) Increased soil moisture and decreased salt content caused by irrigation had the most significant influences on microbial community because the corresponding conditions are substantially improved when an oasis is created.

## Materials and Methods

### Study description and experimental design

The experiments were conducted at the Fukang Station of Desert Ecology, Chinese Academy of Sciences, which is located in the hinterland of the Eurasia continent (44°17′N, 87°56′E). The detailed descriptions of the study site can be found in [11, 20, 21]. This region is covered by sparse halophyte vegetation, which is dominated by *Tamarix ramosissima*, *Reaumuria soongor*,*Nitraria sibirica*,and *Salsola collina*. Due to the limited availability of water, cultivation is only conducted in limited areas, thus oasis farms are generally surrounded by native desert.

A long-term experiment on soil fertility started in 1990. The crop was winter wheat, planted in September each year, and harvested at the end of June or July of the next year. The detailed description for the experimental design can be found in Wang et al. [21]. The treatments selected in this study were: (1) CK (no fertilizer), (2) PK, (3) NK, (4) NP, (5) NPK, (6) NPKR, and (7) NPKM (R: straw residue and M: manure fertilizer). Each treatment had three replicates with a plot size of 33 m^2^. The mean grain yields for the CK, PK, NK, NP, NPK, NPKR, and NPKM treatments were 0.86, 1.06, 2.71, 3.07, 3.71, 3.75, 4.07 t ha^-1^ yr^-1^. The rate of fertilizer application in each treatment is listed in Table 1. The fertilizer application and flood irrigation were described in detail previously [11].

**Table 1:** Fertilizer application rates under different fertilizer treatments in a winter wheat system.

Treatment	Inorganic fertilizer (kg ha^-1^yr^-1^)			Organic fertilizer
	Nitrogen (N)	Phosphorus (P)	Potassium (K)	(t ha^-1^yr^-1^)
CK	0	0	0	0
PK	0	33	50	0
NK	150	0	50	0
NP	150	33	0	0
NPK	150	33	50	0
NPKR	150	33	50	2.5 (Straw)
NPKM	150	33	50	2.5 (Manure)

### Soil sampling, soil properties, microbial biomass, and enzyme activity

After the winter wheat harvest in early July 2011, soil samples were collected both in the oasis receiving different fertilizer applications and the adjacent desert. In the seven treatments of the oasis, five sample points were randomly collected in each of the treatment plots. Soil samples were taken vertically using an auger at the following depth intervals: 0–0.2, 0.2–0.4, 0.4–0.6, 0.6–1, 1–1.5, 1.5–2, 2–2.5, and 2.5–3 m. The undisturbed desert sites (without irrigation and fertilizer application), from which the oasis soils were derived, were sampled using a previously described method [20]. Briefly, three sampling points were placed respectively in bare soil, under shrubs and under grass cover in the native desert, with each one a mixed sample of five locations. The weighted averages of relevant soil parameters were determined according to the area weighting of the soil under plant canopies and in bare land.

The SOC and total N content were measured using a Total Organic Carbon/Total Nitrogen analyzer (Multi C/N 3100, Analytik Jena, Jena, Germany), and total P was determined by acid melt–molybdenum, antimony, and scandium colorimetry. The electrical conductivity (EC) and pH were measured using the conductivity method and potentiometry, respectively (at a soil to water ratio of 1:5). Soil water content was determined by a gravimetric method. Microbial biomass carbon (MBC) was determined by the fumigation-extraction method combined with the Total Organic Carbon/Total Nitrogen analyzer. The activities of five soil enzymes for several metabolic cycles (C, N, P) were determined using a method described by Guan [22]. Invertase activity (EC 3.2.1.26) was determined by 3, 5-dinitrosalicylic acid colorimetry; urease activity (EC 3.5.1.5) was determined by indophenol colorimetry; protease activity (EC: 3.4.4.1) was determined by ninhydrin colorimetry; alkaline phosphatase activity (EC 3.1.3.1.) was determined by phenyl phosphate sodium two colorimetry; and catalase activity (EC 1.11.1.6) was determined by permanganate titration. Phosphate buffers with pH values of 5.5, 6.7, 7.4, 10, 7.8 were used to control pH during the measurements of invertase, urease, protease,alkaline phosphatase, catalase activities, respectively.

### Soil DNA extraction and pyrosequencing

Soil DNA was extracted from approximately 0.5 g of soil (oven dry basis of field-moist soil) using a FastDNA spin kit for soil (MP Biomedicals, Cleveland, OH, USA) according to the manufacturer’s instructions. The quality, quantity, and integrity of the DNA extracts were checked as described by [11]. DNA was PCR-amplified in triplicate using the 515F and 907R primers, which were designed to amplify the hypervariable V3–V4 region of the 16S rRNA gene from bacteria and archaea. Primers were tagged with unique barcodes for each sample. The PCR reactions (Qiagen, Valencia, CA, USA) were conducted as described by [11]. All samples were amplified in triplicate. The A and B adapters required for 454 pyrosequencing were added to specific ends of the PCR products according to [23]. Technical triplicate amplicons were pooled and purified via the gel purification method and quantified using PicoGreen® dye (Invitrogen, Shanghai, China) after they were checked by 1.2% agarose gel electrophoresis. The concentration of purified PCR amplicons was determined, and they were then combined in equimolar ratios into a single tube in preparation for pyrosequencing analysis. Pyrosequencing was performed on a Roche 454 GS FLX Titanium sequencer (Roche Diagnostics Corporation, Branford, CT, USA) according to [23–24]. In this study, pyrosequencing produced approximately 250,000 high-quality sequences with an average read length of about 387 bp.

The 16S rRNA gene sequence reads were processed using ribosomal database project (RDP) pyrosequencing pipeline (http://pyro.cme.msu.edu/) according to [25–27]. Briefly, the readings were aligned by secondary-structure aware infernal aligner and then clustered into Operational Taxonomic Units (OTU) by a custom code that implements the complete-linkage clustering algorithm. The taxonomic identity of each phylotype was determined by the RDP Classifier with an 80% bootstrap score. The diversity indices Shannon (*H*) and Chao1 were estimated using MOTHUR [28] using the greengenes (http://greengenes.lbl.gov/) as the target database.

### Data analysis and statistics

As the topsoil environment was very distinct and the compaction after cultivation occurred at 0–0.6 m according the profile change of the soil bulk density in the study area [20], the total profile was divided into the topsoil (0–0.2 m) and below topsoil (0.2–0.6 m and 0.6–3 m, or 0.2–3 m), in order to make comparisons easier. The weighted means of the relevant parameters below topsoil were obtained by assigning corresponding weight to each depth. Statistical analyses of data were conducted using SPSS 11.5 for Windows (IBM, Endicott, NY, USA). Analysis of variance (ANOVA) and least significant difference (LSD) tests were used to assess the significance of the effects of irrigation, fertilizer application, and soil depth on microbial community and soil properties. The significance level was *P* <0.05.

Canonical correspondence analysis (CCA) can reflect qualitative changes in species composition and maximize the separation of species optima along synthetic axes [29–30]. The analysis was executed in CANOCO 4.5 to determine the relationship between microbial taxa and soil properties, and to assess the effects of irrigation and fertilizer application on the composition and structure of the microbial community. The most influential factor on the microbial community was selected from the environmental variables using the forward selection in CANOCO for Windows and a P value associated with the effect of the environmental variables was given by Monte Carlo test.

## Results

### Soil properties, MBC and enzyme activity

Fertilizer applications not only increased crop yield, but also increased SOC and TN contents in topsoil (0–0.2 m), compared to desert soil or plots with no fertilizer treatment (S1 Table). All treatments in the oasis soil also increased the soil MBC and enzyme activity, including invertase, prolease, catalase, urease, and phosphatase compared to desert soil (S2 Table). The combined application of chemical and organic fertilizer produced the largest changes in soil biochemical properties. In addition, the SOC content decreased below the topsoil in the plots receiving fertilizer treatments, except for the treatments combined with organic fertilizer, and the TN content increased slightly in the deep soil for all treatments in the oasis (S1 Table). Soil EC and pH values significantly decreased and the soil water content significantly increased throughout the soil profile (0–3 m) in the oasis soil (S1 Table). There were no significant differences in the EC, water content and pH value among the treatments within the oasis.

### Effects of irrigation on the abundance of microbial taxon

**Fig 1:**
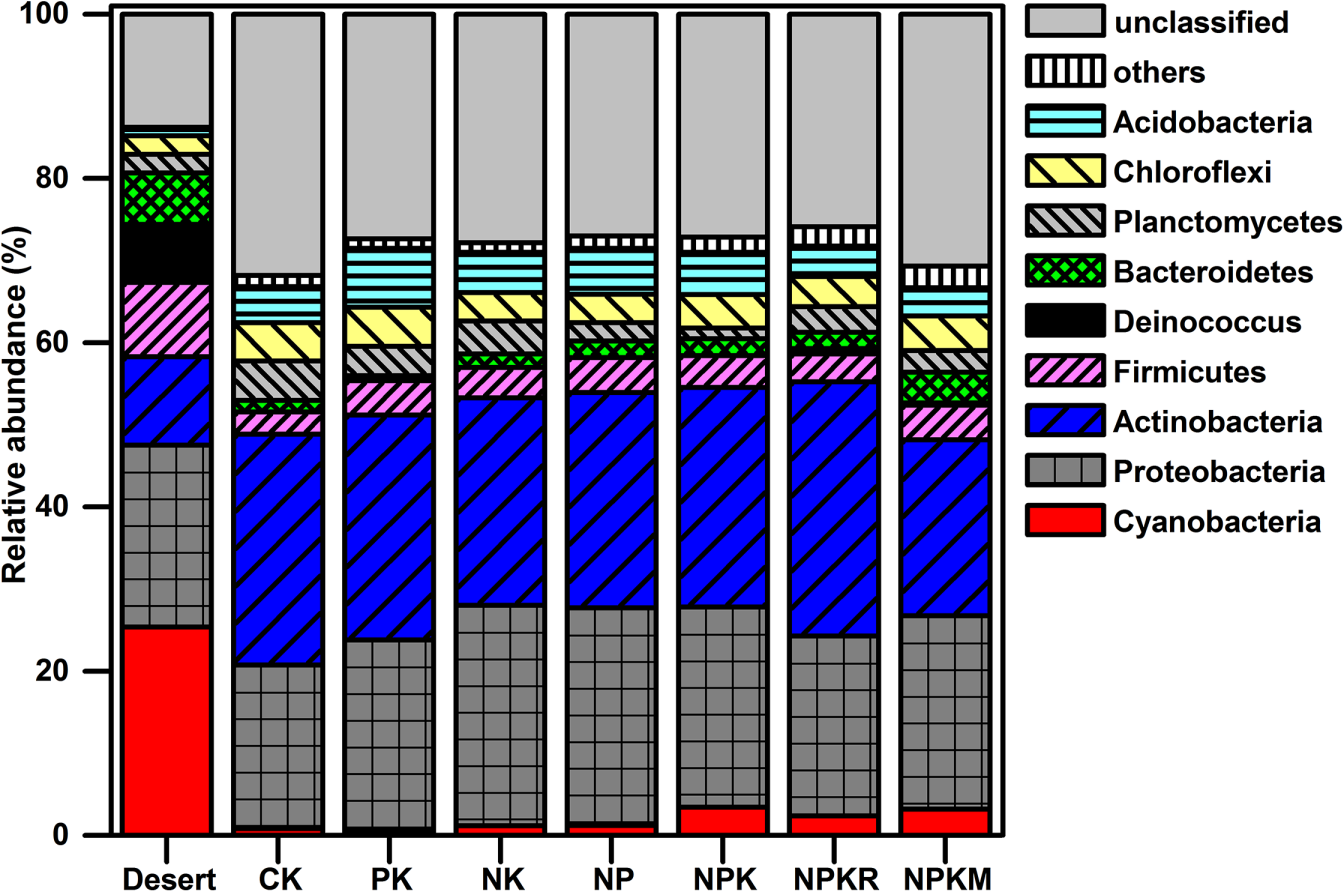
Relative abundances of selected bacterial phyla in topsoil (0–0.2 m) from desert and oasis with different fertilizer treatments. Deinococcus = Deinococcus-Thermus. Desert: the original soil from which the oasis was derived.

**Fig 2:**
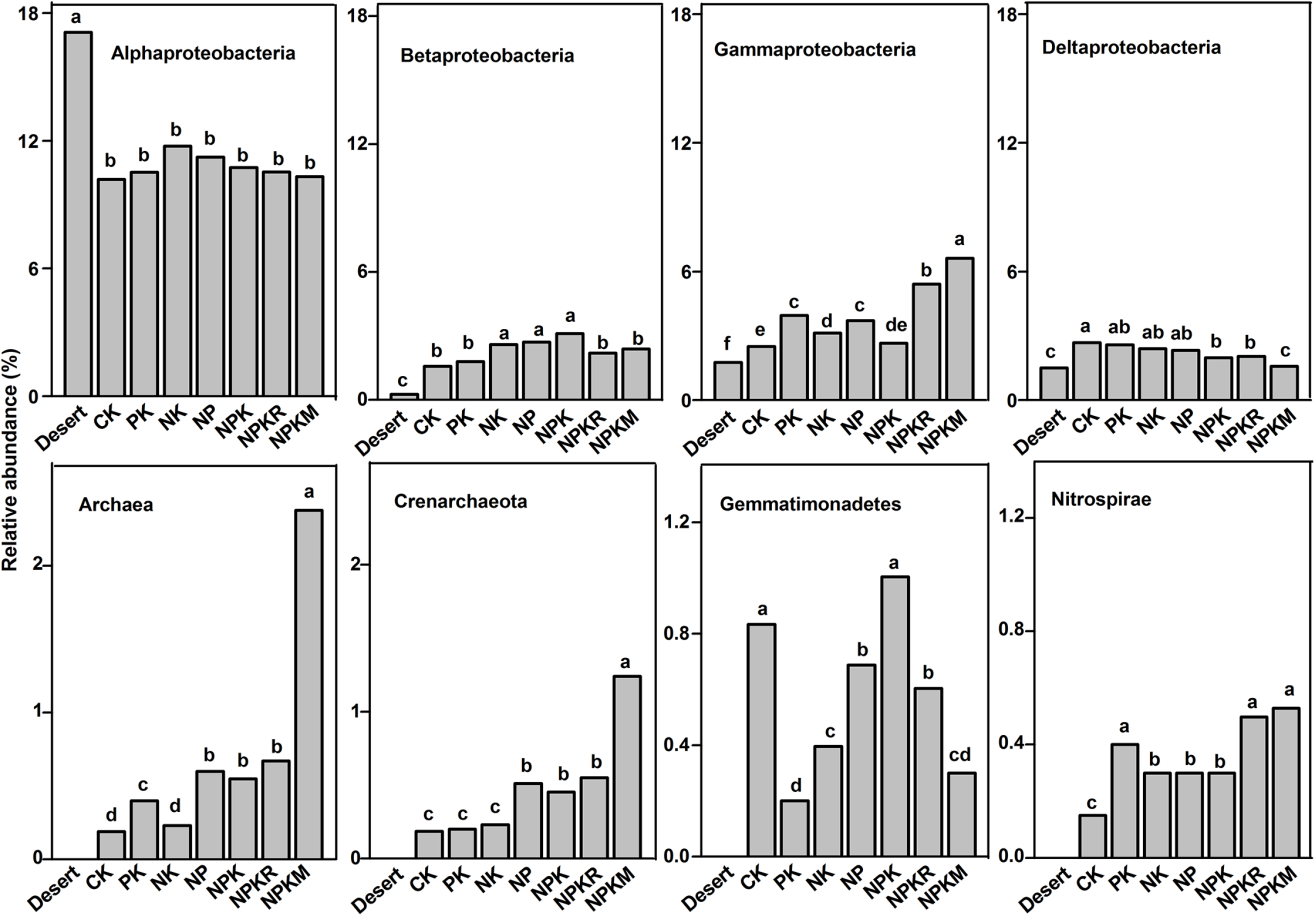
Relative abundances of selected archaea and bacterial taxa in topsoil (0–0.2 m) from desert and oasis with different fertilizer treatments. Desert: the original soil from which the oasis was derived. Different letters at the top of each column indicate that treatment means are significantly different at *p* <0.05.

Irrigation had a significant impact on the soil microbial community in topsoil (0–0.2 m). In the desert topsoil, the most abundant phyla were *Cyanobacteria* (25%) and *Proteobacteria* (22%); whereas in the oasis, the most abundant phyla were *Actinobacteria* (26%) and *Proteobacteria* (24%) (Fig 1). The desert soil had a higher relative abundance of *Cyanobacteria*, *Deinococcus-Thermus*, *Firmicutes*, *Bacteroidetes* (Fig 1), and *Alphaproteobacteria (α-proteobacteria)* (Fig 2); while in the oasis soil, for all treatments there were higher relative abundances of *Actinobacteria*, *Acidobacteria*, *Chloroflexi* (Fig 1), *Gammaproteobacteria (γ-proteobacteria)*, *Betaproteobacteria (β-proteobacteria)*, *Deltaproteobacteria (δ-proteobacteria)*, *Gemmatimonadetes*, *and Nitrospirae* (Fig 2). Moreover, archaea (including *Crenarchaeota*) were only found in oasis soils (Fig 2). Changes were also observed at finer taxonomic divisions (Table 2). Within the *α-proteobacteria*, the proportions of *Rhizobiales*, *Sphingomonadales*, *Rhodobaterales* were lower, but the *Rhodospirillales* were higher in all treatments of the oasis soil, compared to the desert soil. Within the *Actinobacteria*, the relative abundances of the subclasses *Acidimicrobidae* and *Actinobacteridae* were higher, but the *Rubrobacteridae* were lower in the oasis soils than the desert soils. In addition, all of the treatments in the oasis also resulted in higher proportions of the *Xanthomonadales* within the *γ-phaproteobacteria*, *Anaerolineae* within the *Chloroflexi*, *Clostridiales* within the *Firmicutes* and *Gp 6* within the *Acidobacteria*, and lower proportions of the *Bacillales* within the *Firmicutes* than in the desert soil (Table 2).

**Table 2:** Relative abundances of selected bacterial taxa in topsoil (0–0.2 m) from desert and oasis with different fertilizer treatments.

Taxonomic	Name	Desert	CK	PK	NK	NP	NPK	NPKR	NPKM
rank		Relative abundance (%)							
Phylum	*Proteobacteria*								
Class	*α-proteobacteria*								
Order	*Rhizobiales*	6.5 a	3.2 d	3.5 cd	3.8 c	3.8 c	3.5 cd	4.7 b	4.0 c
Order	*Sphingomonadales*	1.3 a	0.4 bc	0.1 c	0.5 bc	0.4 bc	0.1 c	0.7 b	0.3 bc
Order	*Rhodobaterales*	5.8 a	0.2 d	0.2 d	0.7 c	1.4 b	0.7 c	1.0 bc	0.8 c
Order	*Rhodospirillales*	0.5 d	2.5 a	1.8 b	1.8 b	1.2 c	1.1 c	2.7 a	2.9 a
Class	*γ-proteobacteria*								
Order	*Xanthomonadales*	0.1 d	0.4 c	1.2 b	1.7 a	0.8 bc	1.1 b	2.0 a	2.1 a
Order	*Pseudomonadales*	0.0 b	0.3 ab	0.1 b	0.2 ab	0.1 b	0.6 a	0.4 ab	0.3 ab
Phylum	*Actinobacteria*								
Subclass	*Actinobacteridae*	4.8 e	15.2 bc	17.6 bc	12.8 c	18.2 b	18.8 b	22.6 a	8.9 d
Subclass	*Acidimicrobidae*	0.8 d	1.1 d	3.5 a	2.3 bc	1.9 c	2.8 b	1.1 d	0.9 d
Subclass	*Rubrobacteridae*	3.3 a	3.2 a	1.8 c	3.0 ab	1.9 c	2.6 b	2.7 b	0.7 d
Phylum	*Chloroflexi*								
Class	*Anaerolineae*	0.3 c	0.8 b	1.2 ab	1.2 ab	0.8 b	1.2 ab	0.8 b	1.6 a
Phylum	*Firmicuts*								
Order	*Bacillales*	8.8 a	1.6 d	2.6 bc	2.6 bc	2.1 c	2.2 c	3.2 b	2.9 b
Order	*Clostridiales*	0.0 b	0.2 a	0.6 a	0.2 a	0.4 a	0.2 a	0.2 a	0.3 a
Phylum	*Acidobacteria*								
Class	*Gp6*	0.5 c	2.9 a	1.8 b	2.4 a	1.6 b	2.5 a	1.4 b	1.3 b

Different letters within each line indicate that treatment means are significantly different at *p* <0.05.

**Fig 3:**
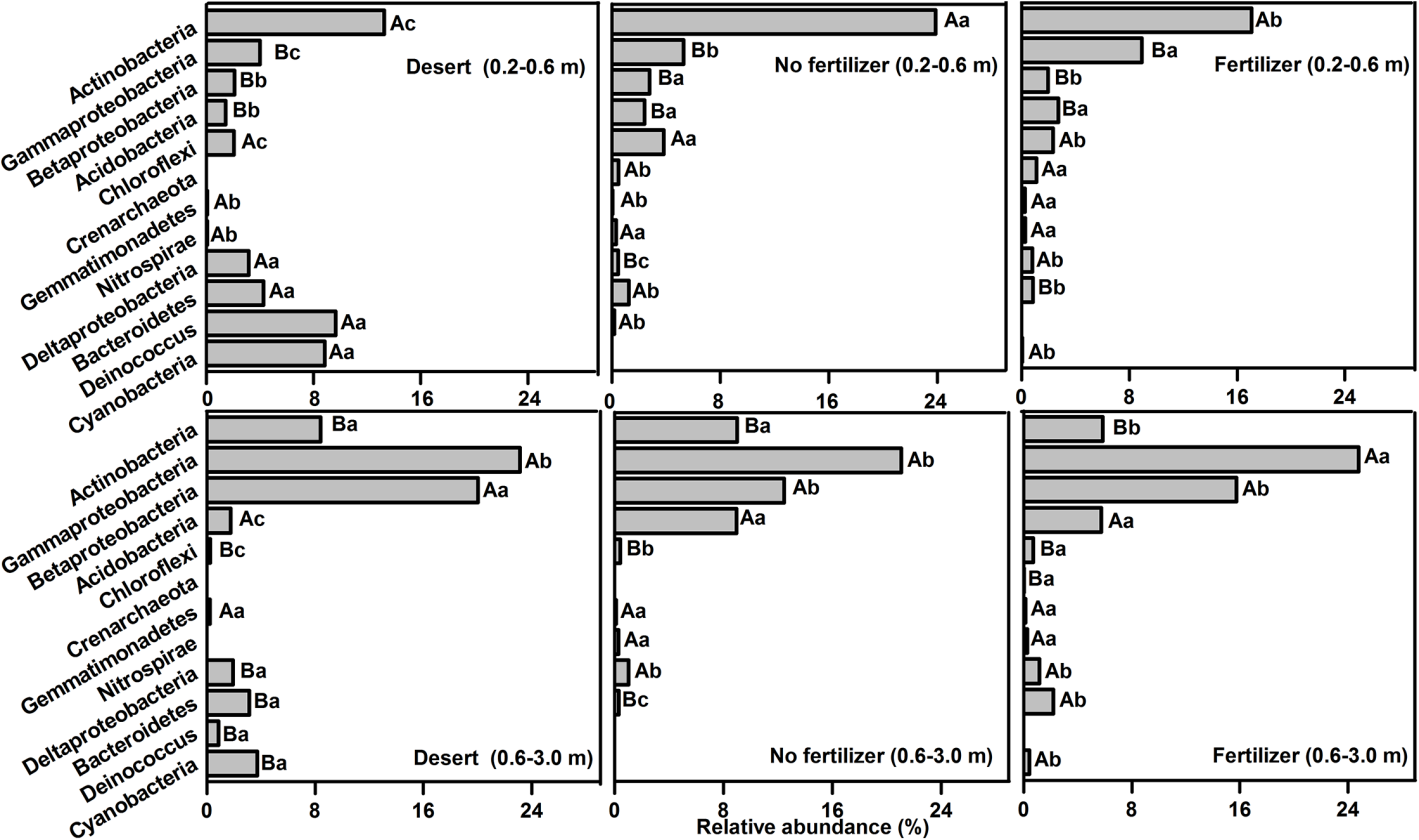
Relative abundances of selected microbial taxa at 0.2–0.6 and 0.6–3 m depths in desert and oasis soils. Desert: the original soil from which the oasis was derived; No fertilizer: the control (CK); Fertilizer: the average values of six fertilizer treatments (PK, NK, NP, NPK, NPKR, and NPKM). Deinococcus = Deinococcus-Thermus. Values at each depth are weighted means. For each microbial taxon, values for different depths within same treatment followed by the same uppercase letter are not significantly different (*p* >0.05); treatment means within same depth followed by the same lowercase letters are not significantly different (*p* >0.05).

As soil depth increased, the relative abundance of *Cyanobacteria* and *Actinobacteria* rapidly decreased, but the abundance of *Proteobacteria*, especially *γ-proteobacteria* significantly increased and became the overwhelmingly dominant population in the deep soil for both desert and oasis (Fig 3). The frequencies and abundances of finer taxonomic divisions (e.g., the orders *Pseudomonadales*, *Oceanospirillales*, and *Enterobacteriales* within the *γ-proteobacteria*) also increased correspondingly with depth (Table 3). Irrigation also had a significant impact on the microbial communities below the topsoil. And the response of most microbial taxa below the topsoil was similar to that in topsoil (Table 3 and Fig 3). However, the responses of some microbial populations were different, e.g., decreases in the proportions of Actinobacteridae, Acidimicrobidae (Table 3), and δ-proteobacteria (Fig 3) below the topsoil, which contrasted with their increases in topsoil, in the oasis comparing to the desert.

**Table 3:** Relative abundances of selected bacterial taxa below topsoil (0.2–3 m) from desert and oasis with different fertilizer treatments.

Phyla	Class/Order	Desert	No fertilizer	Fertilizer
Proteobacteria	*Rhizobiales*	4.8 a	3.7 c	4.3 b
	*Sphingomonadales*	1.6 a	1.6 a	2.0 a
	*Rhodobaterales*	0.9 a	0.2 b	0.4 b
	*Rhodospirillales*	2.2 b	3.0 a	3.4 a
	*Xanthomonadales*	0.7 c	1.2 b	2.5 a
	*Pseudomonadales*	6.3 b	6.6 b	8.0 a
	*Oceanospirillales*	0.5 b	3.1 a	2.8 a
	*Enterobacteriales*	10.4 a	2.8 c	4.9 b
Actinobacteria	*Actinobacteridae*	4.9 a	3.3 b	2.6 c
	*Acidimicrobidae*	0.7 a	0.6 a	0.4 a
	*Rubrobacteridae*	2.0 a	0.8 b	0.7 b
Chloroflexi	*Anaerolineae*	0.1 b	0.4 a	0.4 a
Firmicutes	*Bacillales*	6.8 b	6.8 b	8.3 a
	*Clostridiales*	0.3 b	1.8 a	1.5 a
Acidobacteria	*Gp6*	1.4 b	2.8 a	1.6 b

Desert: the original soil from which the oasis was derived; No fertilizer: the control (CK); Fertilizer: the average values of six fertilizer treatments (PK, NK, NP, NPK, NPKR, and NPKM). Values at the depth are weighted means. Different letters within each line indicate that treatment means are significantly different at *p* <0.05.

### Effects of fertilization on the abundance of microbial taxon

Compared to the treatment without fertilizer (CK), there were significant effects on the microbial community, especially finer taxonomic divisions or non-dominant taxa, in treatments where fertilizer was applied (Fig 2). In the topsoil, fertilizer applications increased the relative abundances of *γ-proteobacteria* (including the order *Xanthomonadales*), *β-proteobacteria*, *Crenarchaeota*, *Nitrospirae*, *Firmicues*, *Rhizobiales*, and *Bacillales* (Fig 2) and slightly decreased that of *δ-proteobacteria* and *Gp 6* (Table 2). Below the topsoil, fertilizer application increased the proportions of *γ-proteobacteria*, *Crenarchaeota*, *Rhizobiales*, and *Bacillales*, but decreased that of *δ-proteobacteria* and *Gp 6* (Table 3 and Fig 3), with higher proportions of the *Enterobacteriales* and *Pseudomonadales* also observed (Table 3). The combined treatments with organic fertilizer produced a larger increase of *γ-proteobacteria* and *Nitrospirae*.

### Correlations between soil properties and microbial taxa

**Fig 4:**
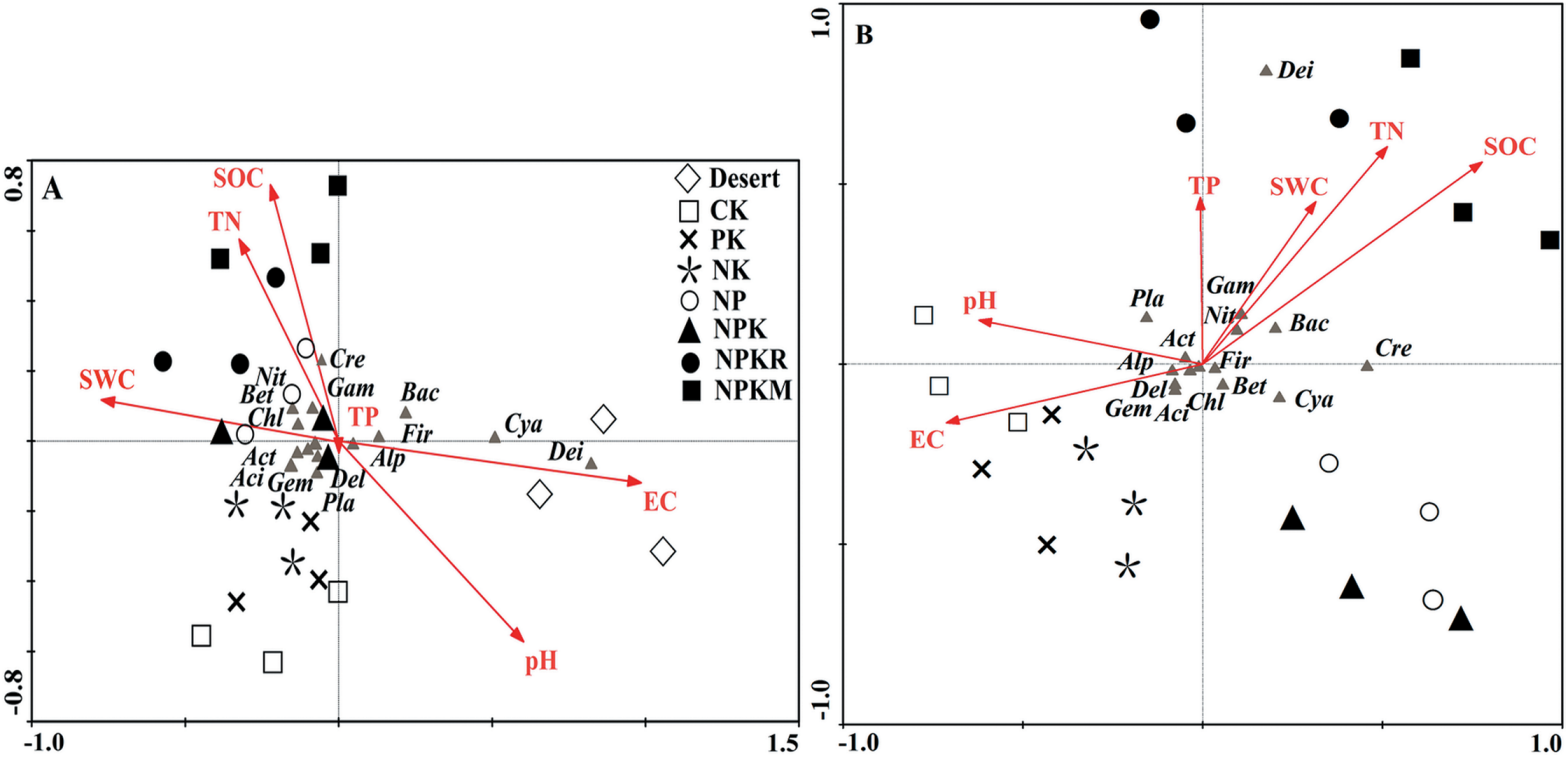
Ordination plots of the results from canonical correspondence analysis (CCA) in topsoil (0–0.2 m) to explore the relationship between microbial populations and soil properties, such as soil organic carbon (SOC), total nitrogen (TN), total phosphorus (TP), soil pH, electric conductivity (EC), and soil water content (SWC) for different fertilizer treatments (CK, PK, NK, NP, NPK, NPKR and NPKM) with (A) and without desert soil (B). Cyanobacteria = Cya, Deinococcus-Thermus = Dei, Actinobacteria = Act, Alphaproteobacteria = Alp, Betaproteobacteria = Bet, Gammaproteobacteria = Gam, Deltaproteobacteria = Del, Acidobacteria = Aci, Firmicutes = Fir, Chloroflexi = Chl, Gemmatimonadetes = Gem, Nitrospirae = Nit, Planctomycetes = Pla, Bacteroidetes = Bac, Crenarchaeota = Cre.

Given that the strongest impact of oasis formation on the microbial community occurred in the topsoil and most community responses were similar throughout the soil profile, we present here only the CCA ordination for topsoil. The ordination plots demonstrate the community differentiations between the desert and oasis soils (Fig 4A), and between different fertilizer applications (Fig 4B). In Fig 4A, the desert soils are centered in areas with low soil moisture and high EC; while the seven treatments of the oasis soils had a relatively concentrated distribution in the opposite area. This indicates that the microbial community structure in the desert soil was completely different from that of the oasis soil, while all treatments in the oasis soil produced roughly similar community structures. However, in the plot without the desert soil (Fig 4B), fertilizer applications exerted significant effects on the microbial community. These treatments were dispersed in different areas of the ordination plot. The CK, PK, and NK treatments were centered along a gradient with relatively high EC, pH, and relatively low SOC and nutrient contents. The combined treatments with organic fertilizers (NPKR and NPKM) were centered on the area with high SOC and nutrient contents and low EC and pH. The NP and NPK treatments were distributed in another area.

According to the forward selection option, soil water content and EC had significant influences on the microbial community structure during oasis formation (both values of *P* were 0.002), while SOC and EC had significant influences during fertilizer application (*P* = 0.012 and 0.04). Most bacterial taxa (e.g., *Cyanobacteria*, *Deinococcus-Thermus*, *Firmicutes*, *Bacteroidetes*, and *α-*, *β-*, *γ-proteobacteria*) exhibited significant correlations with the soil water and EC (*P* <0.05). Some microbial groups (e.g., *Deinococcus-Thermus*, *Acidobacteria*, *β-proteobacteria*, and *Crenarchaeotaaeota*) were associated closely with soil pH (*P* <0.05). Meanwhile, positive correlations were found between certain microbial taxa (e.g., *γ-proteobacteria*, *Nitrospirae*, and *Crenarchaeota*) and SOC, total N contents, MBC, and most enzyme activities (*P* <0.05). These results demonstrated that irrigation had stronger effects on the microbial community than fertilizer application.

## Discussion

### Changes in microbial community as affected by oasis formation

**Fig 5:**
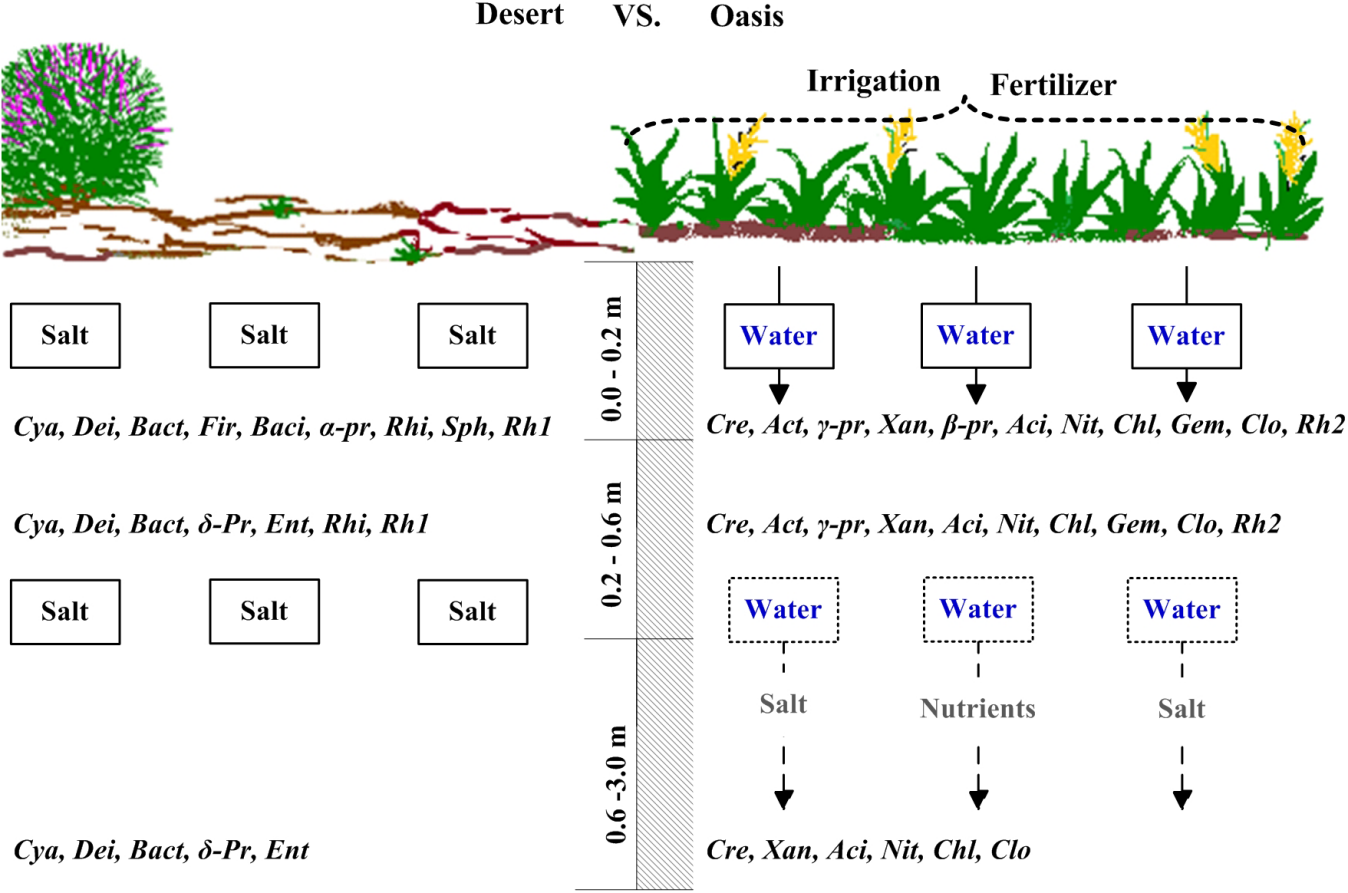
Schematic diagram of the profile changes of dominated microbial community when desert becomes oasis at the southern periphery of the Gurbantonggut Desert. Actinobacteria = Act, Alphaproteobacteria = α-pr, Acidobacteria = Aci, Bacillales = Baci, Bacteroidetes = Bact, Betaproteobacteria = β-pr, Chloroflexi = Chl, Clostridiales = Clo, Crenarchaeota = Cre, Cyanobacteria = Cya, Deinococcus-Thermus = Dei, Deltaproteobacteria = δ-pr, Enterobacteriales = Ent, Gammaproteobacteria = γ-pr, Gemmatimonadetes = Gem, Firmicutes = Fir, Nitrospirae = Nit, Rhizobiales = Rhi, Rhodobaterales = Rh1, Rhodospirillales = Rh2, Sphingomonadales = Sph, Xanthomonadales = Xan.

The results in this study confirmed our basic hypothesis. It demonstrated that cultivation in the desert soil resulted in a strong shift in the microbial community structure throughout the soil profile (0–3 m) (Fig 5). The largest change in the overall microbial community was observed in the topsoil. While converting desert into oasis, the relative abundance of *Cyanobacteria* decreased from 25.4 to 2.5% on average, and that of *Actinobacteria* increased from 10.8 to 26.4% on average. The *Cyanobacteria* were therefore the most abundant group in desert topsoil and the *Actinobacteria* became the most abundant group in oasis topsoil (Fig 1). *Cyanobacteria* participate in both carbon and nitrogen fixation, and generally occur in harsh desert environments [31–32]. In addition to *Cyanobacteria*, other photosynthetic groups (e.g., the *Rhizobiales*, *Sphingomonadales*, and *Rhodobaterales*) were also present in lower proportions in the oasis (Table 2). *Actinobacteria* participate in the decomposition of lignin and chitin [33]. Wheat cover/ lack of light and return of wheat residues to the soil in the oasis may explain above-mentioned community shift. Meanwhile, there were also higher proportions of the *Deinococcus-Thermus*, *α-proteobacteria*, and *Bacillales* present in desert soil (Figs 1 and 2), all of which are extremophilic groups or antagonists, with a strong tolerance to desiccation, radiation, and high levels salinity [34]. The oasis soil had higher proportions of the R-strategy (e.g., *γ-*, *β-proteobacteria*), facultative vegetative (e.g., *Rhodospirillales*) and nitrifying bacteria (e.g., *Nitrospirae*) (Table 2 and Fig 2). Archaea occurred only in oasis soil. There is increasing evidence showing that archaea are involved in ammonia-oxidizing process, and may play significant role in the carbon and nitrogen cycles [24]

Unlike the remarkable change in the predominant group in topsoil, the *γ-proteobacteria* gradually became the overwhelmingly dominant group with the increase of soil depth in both the desert and oasis soils, emphasizing the importance of soil depth as an environmental gradient structuring soil microbial communities. Below the topsoil, most microbial taxa exhibited similar responses to cultivation to that in the topsoil. However, due to the multiple factors involved (e.g., soil moisture, O_2_, salt content, pH, available nutrients, and soil particle composition) and their interactions during oasis formation, some bacterial taxa, e.g., *Actinobacteria* and *δ-proteobacteria*, displayed an inconsistent variation with increased soil depth (Figs 1–3). This inconsistency was also found in some previous studies [13, 35].

Fertilization also had significant impacts on the microbial community structure throughout the soil profile compared to the treatment without fertilizer (CK), but the impacts were less significant than that of irrigation. Generally, fertilizer application had more significant effects on the finer taxonomic divisions or non-dominant taxa. Fertilizer application increased the proportion of *γ-proteobacteria* throughout the profile (Figs 2 and 3). In particular, the *Xanthomonadales* displayed a higher frequency and a greater increase in proportion (Tables 2 and 3), which was similar to the results of previous studies [36–37]. The *Pseudomonadales* and *Enterobacteriales* within the *γ-proteobacteria* were more abundant in deep soil under fertilized treatments, compared to the CK treatment. These bacteria were likely to promote plant and root growth by fixing nitrogen and producing growth hormones [38]. The increase in their abundance may be beneficial to wheat root growth and yield. Moreover, the increase in the proportion of *Bacillales* under fertilizer application may be helpful to prompt plant growth and health, given that many members of the *Bacillales* can secrete various enzymes (e.g., proteases, cellulases and lipases) and are antagonistic toward phytopathogens [14, 39].

This study also demonstrated that desert becoming oasis did not result in higher soil microbial diversity (S3 Table), possibly due to great increase in certain microbial taxa. However, our study indeed showed that oasis formation caused a dramatic shift of soil microbial community in the topsoil and even deep soil. The remarkable changes in soil microbial taxa indicated the improved soil environment and nutritional status in the oasis.

### Correlation between changes in microbial community and soil properties

Microbial communities are strongly influenced by soil properties [35, 40]. Land use or management practices can change the microbial community by changing soil properties, subsequently affecting the microbial diversity and function in soil [41–42]. In this region, extra irrigation was necessary to leach salts due to the natural salinization resulting from low precipitation and high evaporation for crop growth. Therefore, a sharp increase in soil moisture and decrease in EC during oasis formation was evident in the whole profile. As a result, the desert and oasis soils exhibit the largest contrast in soil water and salt statuses. The results also support our second hypothesis that soil water content and EC were the most influential factors driving the changes in the microbial community during agricultural practices in the arid region. For the oasis with different fertilizers (including no fertilizer treatment), which had a similar water and salt status, a roughly similar community structure developed, in contrast to the large community differentiation between the desert and oasis. Therefore, cultivation in the desert drove the shifts in the microbial community mainly through irrigation.

Crop planting in desert region, forming two land covers with the strongest contrast (Fig 5), also exerts a critical influence on soil microbial community. Increased crop yields and the annual return of a large amount of crop residues resulted from fertilizer application provided more substrates to the field for microbial growth. Meanwhile, the increased crop production is likely correlated to increased root growth, providing more exudates/ substrates for soil microbes. Furthermore, the downward leaching of substances from the topsoil could input dissolved organic carbon and nutrients of varying quality and quantity into deep soil during irrigation and fertilizer applications [11]. In these processes, the substances input into the soil profile would inevitably alter soil characteristics, which may favor the growth of certain functional guilds [35] and then introduce new organism into this ecosystem. In the current study, all fertilizer treatments significantly increased the crop yield, which would retain more crop residue with a high C:N ratio into topsoil and provide more substrates to deep soil layers. This led to the substantially altered SOC contents throughout the soil profile during oasis formation (S1 Table). Our statistical analysis demonstrated that the SOC content had the most significant impacts on the structure of soil microbial community for the oasis, which was consistent with the results of many previous studies [36–37, 43–44]. The combined treatments with organic fertilizer produced the greatest increase in SOC content among all treatments due to the 2.5 t ha^-1^yr^-1^ input of organic fertilizer, and also produced a different community structure from the other treatments (Fig 4B).

Cultivation significantly increased the soil moisture and nutrient contents, and decreased the soil salt content and pH. The soil microbial biomass, and the activities of soil enzymes (invertase, prolease, catalase, urease, and phosphatase) related to carbon and nutrient cycling [45] were significantly increased. This demonstrated that cultivation in desert regions offers a favorable soil environment for microbial growth and promotes the degradation and transformation of soil organic matter. Soil enzyme activities are themselves important indicators of biological activity and soil health, and reflect microbial community composition or structure [46–47]. Our results revealed positive correlations between the activity of most enzymes, the SOC and nutrient contents and the increasing proportions of *γ-proteobacteria* and *Nitrospirae* and Archaea during oasis formation. Therefore, oasis formation appears to exert a positive influence on the microbial community structure and composition, especially when chemical fertilizers were applied in combination with organic fertilizers. However, the current study can not determine whether the increased microbial abundance/biomass could result in higher enzymatic rates and SOC content. Clearly, further work is needed to understand the ecology of these microbial taxa.

## Conclusions

Oasis formation remarkably altered the soil microbial community and increased the activity of soil enzymes (invertase, prolease, catalase, urease and phosphatase). The strongest community shift occurred in the topsoil. The predominant phyla in topsoil were shifted from *Cyanobacteria* in the desert to *Actinobacteria* in the oasis, while *proteobacteria*, especially *γ-proteobacteria*, became the predominant group below the topsoil in both the desert and oasis. During oasis formation, the proportion of some photosynthetic groups (e.g., *Cyanobacteria*) and the extremophilic bacterial groups (e.g., *Deinococcus-Thermus*) decreased or even absent, while the proportion of some other microbial populations increased (e.g., *γ-proteobacteria* (including the *Xanthomonadales*), *Nitrospirae*, *Anaerolineae*, and *Chloroflexi*) or occurred (e.g., *Crenarchaeota*) throughout the soil profile (0–3 m). Fertilizer applications also had significant impacts on the microbial community, but the impact was less significant than that of irrigation. Our results suggest that the irrigation, which caused the strong change in soil moisture and salt, was an important driving factor for the community shift during oasis formation; while the effects of fertilizer applications on microbial communities were mainly linked to the altered salt content and especially the SOC. Positive correlations were found between certain microbial taxa (e.g., *γ-proteobacteria*, *Nitrospirae*, and *Crenarchaeota*) and the SOC and total N contents, MBC and soil enzymes.

## Supporting Information

S1 Table Soil properties (organic carbon (SOC), total nitrogen (TN), water content (SWC), electrical conductivity (EC), pH at 0–0.2, 0.2–0.6 and 0.6–3 m depths from desert and oasis with different fertilizer treatments. (DOC)

S2 Table Soil enzyme activities and microbial biomass carbon (MBC) in topsoil (0–0.2 m) from desert and oasis with different fertilizer treatments. (DOC)

S3 Table Soil bacterial diversity richness estimates based on 3% dissimilarity of 16S rRNA gene sequences from desert and oasis with different fertilizer treatments. (DOC)
