# Supplementary material for: Profile Changes in the Soil Microbial Community When Desert Becomes Oasis
Source: PLoS One. 2015 Oct 1;10(10):e0139626. doi: 10.1371/journal.pone.0139626 (PMC4591283; doi:10.1371/journal.pone.0139626)
Supplement: S1 Table — (DOC) [file pone.0139626.s001.doc]

**S1 Table. Soil properties (organic carbon (SOC), total nitrogen (TN), water content (SWC), electrical conductivity (EC), pH at 0–0.2, 0.2–0.6 and 0.6–3 m depths** from desert and oasis with different fertilizer treatments.

| Treatment | 0.0–0.2 m | | | | |
| --- | --- | --- | --- | --- | --- |
|  | SOC  (g kg^-1^) | TN  (g kg^-1^) | SWC  (%) | EC  (mS cm^-1^) | pH |
| Desert | 4.64±0.30 | 0.38±0.03 | 5.0±0.2 | 7.8±1.3 | 9.1±0.2 |
| CK | 4.52±0.19 | 0.43±0.03 | 8.9±0.3 | 0.5±0.1 | 9.0±0.2 |
| PK | 4.76±0.23 | 0.52±0.04 | 9.0±0.4 | 0.6±0.1 | 8.7±0.2 |
| NK | 4.74±0.24 | 0.41±0.02 | 8.9±0.3 | 0.4±0.1 | 8.8±0.2 |
| NP | 5.33±0.33 | 0.44±0.02 | 8.9±0.4 | 0.4±0.1 | 8.7±0.2 |
| NPK | 5.31±0.24 | 0.42±0.03 | 9.1±0.4 | 0.3±0.1 | 8.8±0.2 |
| NPKR | 6.33±0.31 | 0.51±0.04 | 9.2±0.4 | 0.3±0.1 | 8.7±0.2 |
| NPKM | 7.13±0.39 | 0.70±0.05 | 9.2±0.5 | 0.3±0.1 | 8.7±0.1 |
|  | | | | | |
| Treatment | 0.2–0.6 m | | | | |
|  | SOC  (g kg^-1^) | TN  (g kg^-1^) | SWC  (%) | EC  (mS cm^-1^) | pH |
| Desert | 3.21±0.27 | 0.21±0.02 | 10.5±0.9 | 5.2±1.2 | 9.0±0.1 |
| CK | 2.29±0.14 | 0.24±0.03 | 19.6±1.1 | 0.8±0.2 | 8.6±0.1 |
| PK | 2.38±0.17 | 0.19±0.02 | 19.7±1.3 | 1.3±0.3 | 8.5±0.2 |
| NK | 2.62±0.21 | 0.21±0.02 | 19.6±1.2 | 1.0±0.2 | 8.5±0.2 |
| NP | 2.65±0.23 | 0.23±0.03 | 19.8±0.9 | 1.5±0.4 | 8.4±0.2 |
| NPK | 2.68±0.20 | 0.23±0.02 | 19.7±0.8 | 0.8±0.3 | 8.7±0.2 |
| NPKR | 2.77±0.24 | 0.22±0.02 | 19.8±1.1 | 0.5±0.2 | 8.6±0.2 |
| NPKM | 2.69±0.23 | 0.22±0.03 | 19.8±1.2 | 0.6±0.2 | 8.6±0.2 |
|  | | | | | |
| Treatment | 0.6–3.0 m | | | | |
|  | SOC  (g kg^-1^) | TN  (g kg^-1^) | SWC  (%) | EC  (mS cm^-1^) | pH |
| Desert | 1.62±0.05 | 0.11±0.03 | 15.3±1.2 | 2.4±0.5 | 9.7±0.2 |
| CK | 1.63±0.06 | 0.16±0.03 | 22.6±2.1 | 1.2±0.3 | 9.6±0.2 |
| PK | 1.64±0.06 | 0.17±0.03 | 22.9±2.4 | 1.0±0.3 | 9.5±0.1 |
| NK | 1.50±0.05 | 0.17±0.02 | 23.2±2.0 | 1.1±0.2 | 9.6±0.1 |
| NP | 1.51±0.05 | 0.16±0.02 | 23.4±2.1 | 1.3±0.3 | 9.6±0.1 |
| NPK | 1.52±0.06 | 0.15±0.02 | 23.3±1.8 | 1.1±0.3 | 9.7±0.3 |
| NPKR | 1.72±0.06 | 0.18±0.03 | 23.6±2.4 | 0.7±0.2 | 9.6±0.2 |
| NPKM | 1.65±0.05 | 0.18±0.02 | 23.8±2.4 | 0.7±0.2 | 9.6±0.2 |
