# Supplementary material for: Profile Changes in the Soil Microbial Community When Desert Becomes Oasis
Source: PLoS One. 2015 Oct 1;10(10):e0139626. doi: 10.1371/journal.pone.0139626 (PMC4591283; doi:10.1371/journal.pone.0139626)
Supplement: S2 Table — (DOC) [file pone.0139626.s002.doc]

**S2 Table.** **Soil enzyme activities and microbial biomass carbon (MBC) in topsoil (0-0.2 m) from desert and oasis with different fertilizer treatments.**

| Treatment | Invertase | Urease | Catalase | Protease | Phosphatase | MBC |
| --- | --- | --- | --- | --- | --- | --- |
|  | (mg g^-1^ d^-1^) | (mg g^-1^ d^-1^) | (mL g^-1^ d^-1^) | (mg g^-1^ d^-1^) | (mg g^-1^ d^-1^) | (mg g^-1^) |
| Desert | 3.5±0.8 | 0.20±0.03 | 2.0±0.4 | 0.2±0.1 | 0.4± 0.1 | 3.0±0.5 |
| CK | 5.7±0.7 | 0.40±0.04 | 4.1±0.5 | 0.4±0.1 | 0.6±0.1 | 4.2±0.2 |
| PK | 5.8±0.6 | 0..43±0.04 | 3.5±0.4 | 0.5±0.1 | 0.6±0.1 | 4.4±0.3 |
| NK | 8.3±1.1 | 0.67±0.06 | 4.0±0.4 | 0.6±0.2 | 0.8±0.2 | 4.1±0.3 |
| NP | 9.3±1.2 | 0.65±0.05 | 3.8±0.3 | 0.7±0.2 | 0.7±0.2 | 4.8±0.4 |
| NPK | 8.2±1.1 | 0.70±0.05 | 3.9±0.4 | 0.7±0.2 | 0.7±0.1 | 4.5±0.4 |
| NPKR | 12.1±1.8 | 0.74±0.06 | 4.0±0.5 | 0.8±0.2 | 0.7±0.1 | 5.7±0.6 |
| NPKM | 10.4±1.2 | 0.72±0.05 | 3.8±0.4 | 0.9±0.3 | 0.8±0.2 | 5.6±0.6 |
