# Supplementary material for: Profile Changes in the Soil Microbial Community When Desert Becomes Oasis
Source: PLoS One. 2015 Oct 1;10(10):e0139626. doi: 10.1371/journal.pone.0139626 (PMC4591283; doi:10.1371/journal.pone.0139626)
Supplement: S3 Table — (DOC) [file pone.0139626.s003.doc]

**S3 Table. Soil bacterial diversity richness estimates based on 3% dissimilarity of 16S rRNA gene sequences from desert and oasis with different fertilizer treatments.**

|  | Desert | CK | PK | NK | NP | NPK | NPKR | NPKM |
| --- | --- | --- | --- | --- | --- | --- | --- | --- |
| Shannon (*H)* | 5.83 | 5.98 | 5.02 | 5.38 | 5.59 | 5.25 | 5.76 | 5.85 |
| Chao 1 | 2816 | 3275 | 2292 | 2731 | 2753 | 2701 | 2820 | 3218 |
